# Management of Pest Insects and Plant Diseases by Non-Transformative RNAi

**DOI:** 10.3389/fpls.2019.01319

**Published:** 2019-10-25

**Authors:** Deise Cagliari, Naymã P. Dias, Diogo Manzano Galdeano, Ericmar Ávila dos Santos, Guy Smagghe, Moisés João Zotti

**Affiliations:** ^1^Laboratory of Molecular Entomology, Department of Crop Protection, Federal University of Pelotas, Pelotas, Brazil; ^2^Sylvio Moreira Citrus Center, Campinas Agronomic Institute (IAC), Cordeirópolis, Brazil; ^3^Department of Plants and Crops, Ghent University, Ghent, Belgium

**Keywords:** RNAi, non-transgenic RNAi, RNA-based products, gene silencing, pest insects, plant diseases

## Abstract

Since the discovery of RNA interference (RNAi), scientists have made significant progress towards the development of this unique technology for crop protection. The RNAi mechanism works at the mRNA level by exploiting a sequence-dependent mode of action with high target specificity due to the design of complementary dsRNA molecules, allowing growers to target pests more precisely compared to conventional agrochemicals. The delivery of RNAi through transgenic plants is now a reality with some products currently in the market. Conversely, it is also expected that more RNA-based products reach the market as non-transformative alternatives. For instance, topically applied dsRNA/siRNA (SIGS – Spray Induced Gene Silencing) has attracted attention due to its feasibility and low cost compared to transgenic plants. Once on the leaf surface, dsRNAs can move directly to target pest cells (e.g., insects or pathogens) or can be taken up indirectly by plant cells to then be transferred into the pest cells. Water-soluble formulations containing pesticidal dsRNA provide alternatives, especially in some cases where plant transformation is not possible or takes years and cost millions to be developed (e.g., perennial crops). The ever-growing understanding of the RNAi mechanism and its limitations has allowed scientists to develop non-transgenic approaches such as trunk injection, soaking, and irrigation. While the technology has been considered promising for pest management, some issues such as RNAi efficiency, dsRNA degradation, environmental risk assessments, and resistance evolution still need to be addressed. Here, our main goal is to review some possible strategies for non-transgenic delivery systems, addressing important issues related to the use of this technology.

## Introduction

From the earliest days of agriculture, mankind cultivated the land to feed their descendants, allowing for an increase in population growth over the years. Now, thousands of years later, modern agriculture is facing one of its biggest challenges: How are we going to produce food in a profitable, efficient, and sustainable way to feed about 10 billion people by 2050? Agricultural productivity has been facing several issues that limit crop production below its maximum potential, namely damage by insects, diseases, and competition with weeds. For instance, insects are responsible for 20 to 40% of yield loss (Oerke, 2006). Moreover, researchers expect a 10 to 25% increase in insect damage per global temperature degree increment in the next years, with the main problems being in the temperate regions (Deutsch et al., 2018).

In an attempt to reduce the damage caused by pests, growers rely heavily on synthetic chemicals, which have been developed and applied since the 1930s. Pesticides allowed growers to increase production, improve product quality, and yield better profits. In 2012, growers around the world spent nearly $56 billion on pesticides, amounting to nearly 6 billion pounds of chemicals used in 2011 and 2012 (Atwood and Paisley-Jones, 2017). The high amount of chemicals used every year is leading to an increase in pesticide resistance, with a significant increase in resistance cases in insects (APRD 2019, https://www.pesticideresistance.org/search.php).

Modern agriculture is now entering the third green revolution, based on the significant progress in the use of reverse genetics to elucidate gene function and applying this knowledge in pest management. Major progress was made by Fire and Mello in 1998 by elucidating the gene-silencing mechanism in eukaryotic organisms named as RNA interference (RNAi) (Fire et al., 1998). RNAi, also known as Post Transcriptional Gene Silencing (PTGS), is a natural mechanism of gene regulation and is a defense system against viruses in eukaryotic cells (Hannon, 2002; Baum and Roberts, 2014) by degradation of the messenger RNA (mRNA) and reduction or complete elimination of the expression of a target gene (Fire et al., 1998).

Since the elucidation of the gene-silencing mechanism in eukaryotic organisms, significant advances have been made related to the use of this technique in the management of insect pest (Gordon and Waterhouse, 2007; Price and Gatehouse, 2008; Huvenne and Smagghe, 2010; de Andrade and Hunter, 2016; Joga et al., 2016; San Miguel and Scott, 2016; Zotti et al., 2017) and plant diseases (Fu et al., 2005; Koch et al., 2013; Jahan et al., 2015; Koch et al., 2016; Wang et al., 2016b; Tiwari et al., 2017; Wang et al., 2017). Recently, the development by Bayer and approval of the SmartStax PRO maize carrying event MON87411 in Canada (2016) and the United States of America (USA) (2017) to control *Diabrotica virgifera virgifera* is considered a milestone in the use of RNAi technology in agriculture (Head et al., 2017). This technology is now available to growers as a tool for pest management. Delivery of double-stranded RNA (dsRNA) through this RNAi transformative approach (*i.e.,* transgenic plants) is a promising way to induce gene silencing in a specific pest (Baum and Roberts, 2014; Ghag, 2017), however it is not practical to every target organism or crop. Also, one of the key disadvantages of transgenic plants and seeds rely on regulatory approval, which takes years and is costly.

We are witnessing a constant decrease in the cost of dsRNA production together with an increased attraction from companies towards the development of improved dsRNA production techniques. It is therefore believed that non-transformative RNAi will soon reach the market (San Miguel and Scott, 2016; Cagliari et al., 2018; Mat Jalaluddin et al., 2018; Dubrovina and Kiselev, 2019). However, some issues are still hindering the development of non-transformative RNA-based products. In this paper, we aim to present the successful studies using non-transformative delivery systems and discuss limitations and possible solutions.

## RNAi Mechanism: From RNA Delivery to Gene Silencing

RNAi-based gene silencing can be triggered in the target organism by the supply of RNAs in two forms: (1) the delivery of dsRNA molecules or (2) the direct delivery of small RNAs (sRNAs). Currently, there are two major classes of sRNAs acting on the RNAi pathway: microRNAs (miRNAs) and small-interfering RNAs (siRNAs). MiRNAs are endogenously derived and involved in the regulation of gene expression, while siRNAs can be of exogenous origin from viruses or artificial supply (Preall and Sontheimer, 2005; Matranga and Zamore, 2007), or of endogenous origin from transposons (Lippman and Martienssen, 2004; Golden et al., 2008). It is known that, in most cases, insects take up dsRNAs longer than 50 bp but not sRNAs (Feinberg and Hunter, 2003; Saleh et al., 2006; Ivashuta et al., 2015), although some studies have shown that sRNA can trigger gene silencing (Borgio, 2010; Gong et al., 2013). By contrast, fungi and plants take up both dsRNAs and sRNAs (Koch et al., 2016; Wang et al., 2016b), suggesting that these organisms have a different uptake mechanism (Wang et al., 2017).

**Figure 1:**
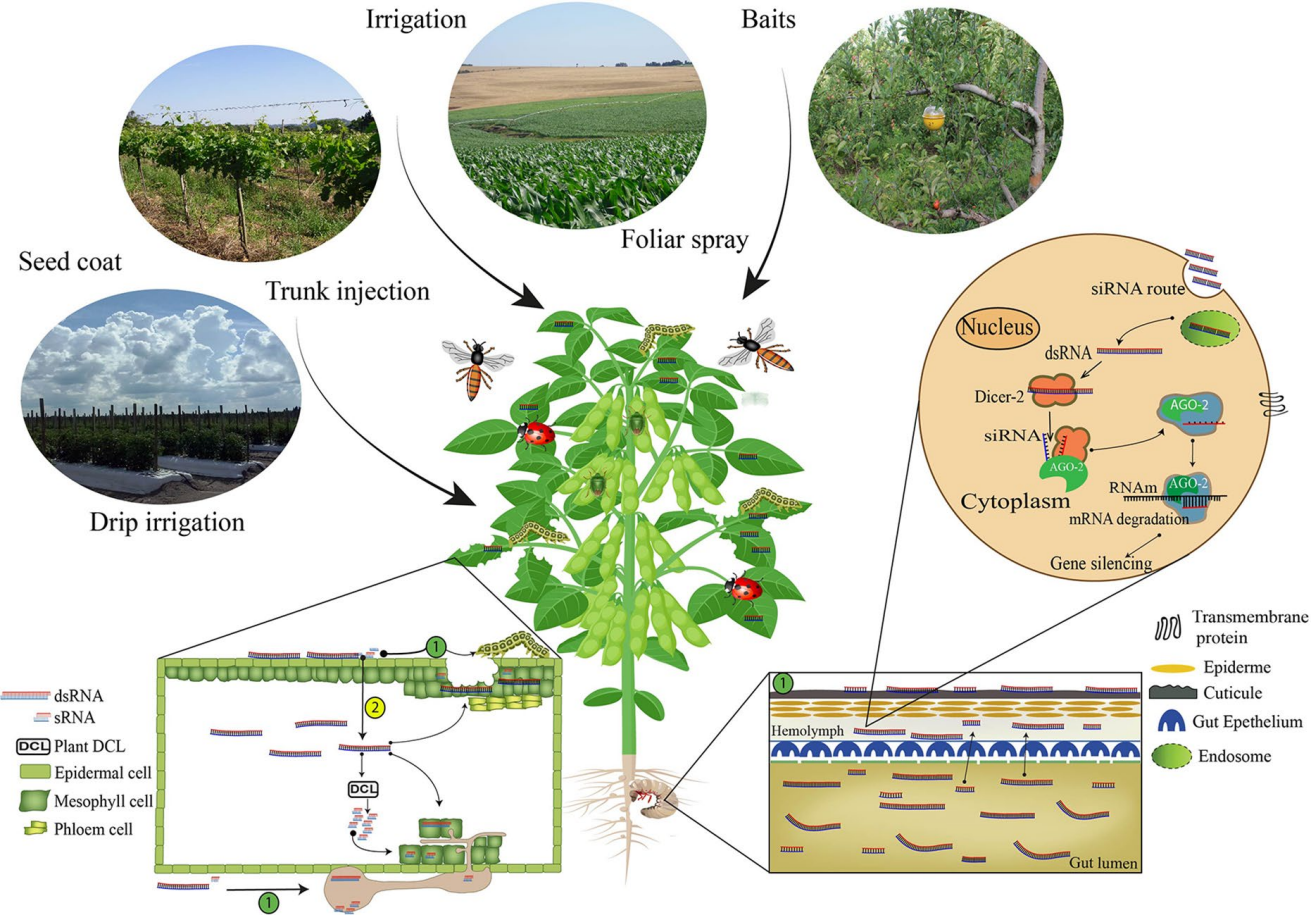
Non-transformative delivery strategy routes for RNAi-based gene silencing induction. The first step to achieve successful RNAi-based gene silencing results *via* non-transformative approaches is the selection of the RNAs (dsRNA or siRNA) delivery strategy: Foliar spray, trunk injection, irrigation, drip irrigation, seed coat, baits, and powder or granules for soil applications. Once the RNAs are delivered, the insects and pathogens need to internalize the RNAs molecules, and this process can occur (1) directly or (2) indirectly. The direct uptake occurs when the organisms get in contact with the RNAs molecules during application or feed on tissues containing the RNA molecules on the surface. However, when the RNA molecules are absorbed, translocated in the plant vascular system and taken up by the organism (Koch et al., 2016), the process is classified as indirect uptake (Cagliari et al., 2018). Inside the organism system, the cell uptake of dsRNA can be mediated by transmembrane channel proteins such as sid-1 (Feinberg and Hunter, 2003; Aronstein et al., 2006; Kobayashi et al., 2012) or endocytosis (Saleh et al., 2006; Ulvila et al., 2006; Cappelle et al., 2016; Pinheiro et al., 2018; Vélez and Fishilevich, 2018). The RNAi-based gene silencing depends on the release at cellular levels of dsRNA or siRNA molecules (Carthew, 2009; Zotti and Smagghe, 2015). When dsRNAs are unloaded in the cytoplasm, these molecules are processed into siRNA fragments by an enzyme called Dicer 2 (DCR-2) (Meister and Tuschl, 2004; Tomari et al., 2007). The siRNA fragments are then incorporated into the RISC complex (RNA-induced Silencing Complex), which contains the Argonaute 2 (AGO-2) protein (Matranga et al., 2005; Miyoshi et al., 2005; Ketting, 2011), and, in a sequence-specific manner, bind to a complementary messenger RNA (mRNA), cleave it, prevent protein formation (Agrawal et al., 2003; Huvenne and Smagghe, 2010), and thus affect target organism survival.

Once RNA molecules are delivered in the field (*i.e., via* transgenic plant, foliar spray, or trunk injection), they need to enter the cell of a target organism to trigger gene silencing. This process can occur through (a) direct or (b) indirect uptake (**Figure 1**). Direct uptake occurs when the RNA molecules are taken up through topical contact or feeding on plant tissues. By contrast, indirect uptake of RNA molecules involves first entering into the plant vascular system and then uptake by the insect/pathogen (Cagliari et al., 2018). The uptake process in the target pest is closely related to the delivery strategy, as demonstrated in several studies (**Table 1**).

**Table 1:** Non-transformative delivery approaches and the relation between the organism location on the plant and the initial RNA uptake process.

Non-transformative delivery system	Insect/Pathogen location	RNA uptake process by the target organism	Reference
Soil drench; Drip irrigation; Irrigation	Roots; Stem; Leaves	Direct/Indirect	(Hunter et al., 2012; Li et al., 2015; Ghosh et al., 2017)
Seed coat or powder/granules	Roots; Stem	Direct/Indirect	–
Sprayable products	Stem; Leaves; Fruits/seeds	Direct/Indirect	(Hunter et al., 2012; Weiberg et al., 2013; de Andrade and Hunter, 2016; Wang et al., 2016b; Koch et al., 2016; San Miguel and Scott, 2016; Gogoi et al., 2017; Mitter et al., 2017b; McLoughlin et al., 2018; Niehl et al., 2018; Song et al., 2018; Gu et al., 2019; Worrall et al., 2019)
Trunk injection	Roots; Stem; Leaves; Fruits/seeds	Indirect	(Dalakouras et al., 2018; Hunter et al., 2012; Berger and Laurent, 2019)
Baits	Fruits	Direct	–

Successful direct uptake *via* topical application has already been reported in different organisms (Pridgeon et al., 2008; El-Shesheny et al., 2013; Killiny et al., 2014). Zheng et al. (2019) reported that a dsRNA formulated in a nanocarrier plus a detergent was able to cross the cuticle in *Aphis glycines*, leading to a reduction of 95.4% in gene expression. Also, indirect uptake of dsRNA has been reported in some insects (Ghosh et al., 2017) and pathogens (Koch et al., 2016). However, there are some limitations related to the indirect uptake process, such as efficiency of translocation of the RNA molecules inside the plant vascular system and dsRNA processing by the plant RNAi machinery. Although it is known that RNAs can move through the plant vascular systems and plant cells (Melnyk et al., 2011; Molnar et al., 2011; Gogoi et al., 2017), some results have shown inefficient translocation of these molecules inside the plant vascular system. For example, in *Malus domestica* and *Vitis vinifera* treated with dsRNA and siRNA, the RNA molecules spread from treated to non-treated tissues but were restricted to the xylem vessels (Dalakouras et al., 2018). This study also found that in *Nicotiana benthamiana*, siRNA molecules were not efficiently translocated. In pathogens, studies on gene silencing found evidence of external dsRNA processing into siRNAs (Koch et al., 2016; Konakalla et al., 2016; Mitter et al., 2017a). In *Hordeum vulgare*, dsRNA locally applied on detached leaves was taken up by plant cells, translocated through the vascular system, and processed into siRNAs by the plant Dicer enzyme, resulting in the inhibition of *Fusarium graminearum* growth in local and distal unsprayed leaves (Koch et al., 2016). In this study, the dsRNA molecules were found in xylem and phloem parenchymal cells, companion cells, mesophyll cells, and in trichomes and stomata, showing that the plant cells took up the dsRNAs. In citrus and grapevine plants treated with dsRNA, siRNAs were found in plants up to three months after treatment, indicating that the dsRNA was processed by the plant RNAi machinery (Hunter et al., 2012).

In some organisms, the process of dsRNA uptake by the cells can be mediated by transmembrane channel proteins such as sid-1 (Feinberg and Hunter, 2003; Aronstein et al., 2006; Kobayashi et al., 2012) or endocytosis (Saleh et al., 2006; Ulvila et al., 2006; Cappelle et al., 2016; Pinheiro et al., 2018; Vélez and Fishilevich, 2018). Recently, in *Drosophila*, scientists elucidated the involvement of nanotube-like structures, which mediate cell-to-cell trafficking of sRNA and RNAi machinery components, allowing gene silencing in cells and tissues distant from the uptake point (Karlikow et al., 2016). However, the uptake system of RNA varies among insects, even within the same order (Vélez and Fishilevich, 2018), resulting in variations in the efficiency of gene silencing.

Although a number of RNAi pathways use dsRNAs to generate sRNAs (*i.e.* microRNA and siRNA) (Bernstein et al., 2001; Ketting, 2011), in insects and fungi the siRNA pathway is known to be activated due to the presence of dsRNA molecules or a direct siRNA supply (Carthew, 2009; Zotti and Smagghe, 2015). Once inside the cell, dsRNAs are processed into siRNA fragments of ∼20 base pairs (bp) in length by a ribonuclease III enzyme called Dicer 2 (DCR-2) (Meister and Tuschl, 2004; Tomari et al., 2007). The siRNA fragments are then incorporated into the RISC complex (RNA-induced Silencing Complex), which contains the Argonaute 2 (AGO-2) protein (Matranga et al., 2005; Miyoshi et al., 2005; Ketting, 2011). After unloading the non-incorporated passenger strand, the complex binds in a sequence-specific manner to the complementary mRNA, cleaving it, and preventing translation to protein (Agrawal et al., 2003; Huvenne and Smagghe, 2010).

The spread of the RNAi signal in the organism can be cell-autonomous or non-cell-autonomous (Whangbo and Hunter, 2008; Huvenne and Smagghe, 2010). In cell-autonomous RNAi, silencing effects are observed only in the cells directly exposed to the dsRNA (Huvenne and Smagghe, 2010). By contrast, in non-cell-autonomous RNAi, the silencing effects are detected in exposed and non-exposed cells, even in different tissues (Whangbo and Hunter, 2008). Non-cell-autonomous RNAi is classified as environmental RNAi, a concept describing all processes in which dsRNA/siRNA are taken up from the environment by a tissue/cell and spread from one cell to another, or from one tissue type to another, through systemic RNAi (Huvenne and Smagghe, 2010). In plants, fungi, and the nematode *Caenorhabditis elegans*, the RNA-dependent RNA polymerase (RdRp) enzyme synthesizes secondary siRNAs by targeting single-stranded RNA molecules (ssRNA) and synthesizing a second strand, consequently generating dsRNA molecules and producing a systemic spread of the RNAi signaling (Zotti et al., 2017). The systemic nature of RNAi has already been observed in insects (Tomoyasu et al., 2008; Whyard et al., 2009; Wynant et al., 2012), however, the systemic RNAi mechanism is still unknown in this group. What is known about this process so far is that the dsRNA/siRNA spread from one cell to another cell or tissue is highly dependent on the cell’s ability to take up the dsRNA or siRNA molecules (Vélez and Fishilevich, 2018), or on mediation through nanotube-like structures (Karlikow et al., 2016).

## Why Use Non-Transformative Delivery Strategies for Pest Management?

RNAi in crop protection can be achieved by plant-incorporated protectants (PIPs) through plant transformation (*i.e.*, transgenic plants) or by non-transformative strategies through a spray-induced gene silencing (SIGS) process (**Table 2**). Regardless of the delivery strategy, the use of RNA-based products to confer plant protection against insects and pathogens is a potential alternative to conventional pesticides (Koch et al., 2016).

**Table 2:** Different features affecting the development of RNAi-based products: Transformative vs. Non-transformative methods.

Feature	Strategy	
	Transformative	Non-transformative¹
Development time	High	Low²
Development costs	High	Low
Feasibility according to culture	Unviable for some plant species	Viable for all cultures¹
Delivery of sRNA	Continuous	Transient
Feasibility according to the pest	Most pests can be targeted due to continuous dsRNA supply feature	Not all pests can be targeted due to recalcitrant features
Development of resistance	High	Low
Regulatory process	Extensive	Simple
Acceptance by consumers	Low	High

¹Non-transformative delivery approaches: foliar application, trunk injection, and irrigation water among others; ²Non-transforative strategy compared to transformative strategy.

**Figure 2:**
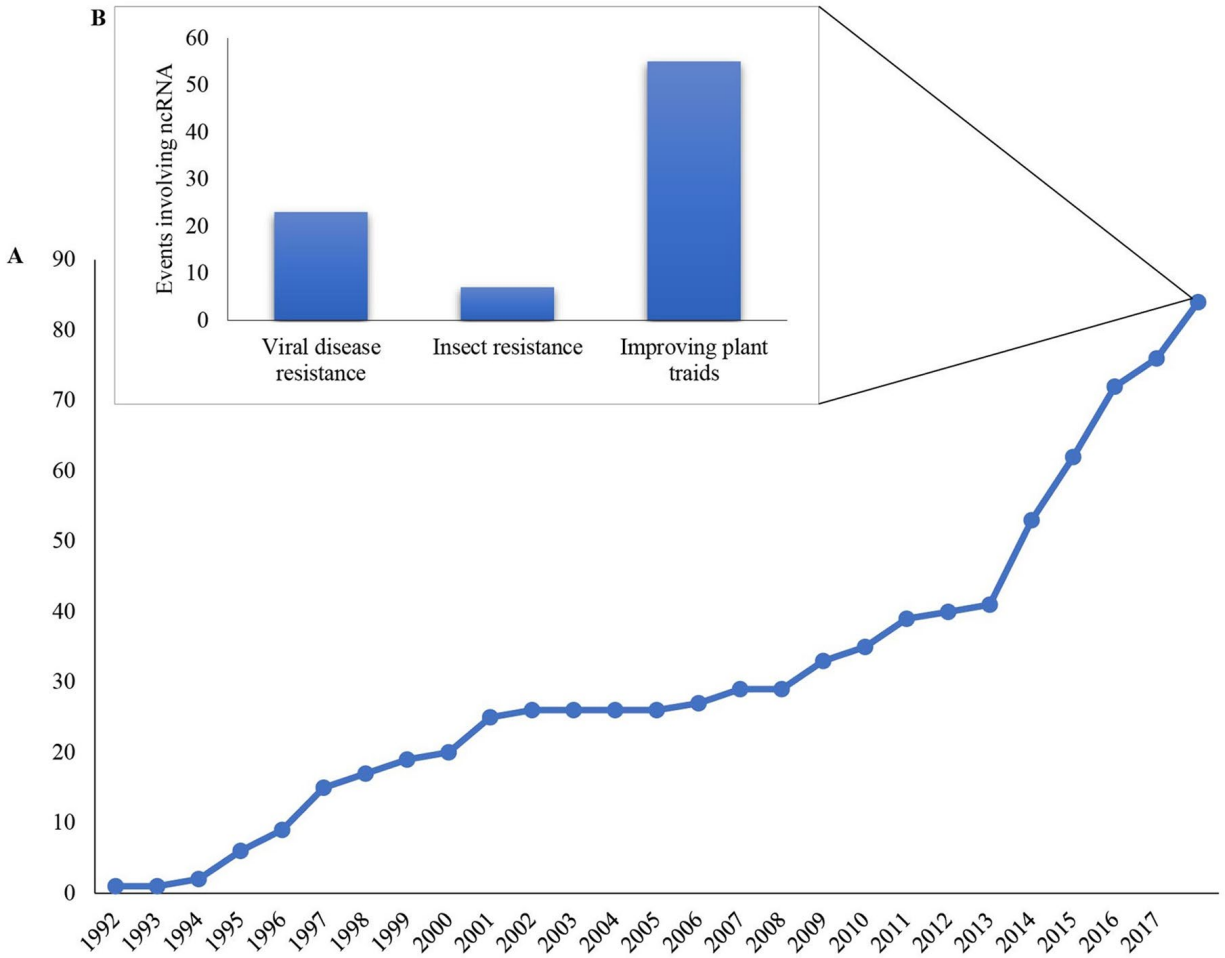
Accumulated, approved genetically modified events based on non-coding RNA (ncRNA) worldwide for cultivation since 1992. **(A)** Total approved ncRNA GM events worldwide since the first ncRNA approved event in 1992; **(B)** Number of ncRNA GM events according to the desired features. The data used to make the graphics were compiled from the GM Approval Database at the International Service for the Acquisition of Agri-Biotech Applications (ISAAA) (http://www.isaaa.org/gmapprovaldatabase/default.asp).

Currently, approved RNAi-based GM plants are based on ncRNA (non-coding RNA) to control insects (8%) and diseases (27%) or to improve specific plant traits (65%), with an increase in approved events over the last years (**Figure 2**). In 2016, the first transgenic RNAi crop (SmartStax PRO maize) combining *Bt* (*Bacillus thuringiensis*) toxin with RNAi for insect control was released for cultivation in Canada and a year later in the USA (Head et al., 2017). In general, the delivery of dsRNA in the field is facilitated by the use of GM plants, however, this strategy still cannot be adopted in all plants/crops due to the high cost of production and the long time for development. For instance, the commercial availability of “HoneySweet,” a cultivar resistant to the *Plum pox virus* (PPV), took 20 years to reach the market (Scorza et al., 2013). Also, there are no established transformation protocols for most of the cultivated plants, which may cause a substantial delay in the development of RNAi-based GM plants (Mitter et al., 2017b). Therefore, alternative strategies for the delivery of RNA biopesticides are necessary and could provide alternative ways to use this technology in the field. Given that non-transgenic RNAi-based products would silence genes without introducing hereditary changes in the genome, it is expected that they will not be regulated as GM products, thereby reducing the time and processes for their release to use as well as potentially improving public acceptance (Cagliari et al., 2018).

Studies are being carried out prospecting non-transformative approaches to control insects, diseases, nematodes, and weeds, and it is expected that RNAi-based products will reach the market in the form of sprayable products for foliar application, trunk injection, root dipping, or seed treatment as direct control agents (Zotti and Smagghe, 2015; San Miguel and Scott, 2016; Zotti et al., 2017; Cagliari et al., 2018; Berger and Laurent, 2019; Dubrovina and Kiselev, 2019). The RNA-based new generation of biopesticides could circumvent the technical limitation of plant transformation and the public’s concerns about GM plants, providing an easy-to-use tool for crop production and storage, as well as an environmentally friendly pest management strategy (Wang et al., 2017; Zotti et al., 2017). Furthermore, RNA-based biopesticides could be efficiently designed to target multiple insects or pathogen species.

The development of resistance is an important point regarding the use of non-transformative delivery strategies. Although dsRNAs longer than 200 nucleotides result in many siRNAs post-cleavage, maximizing the RNAi response and reducing resistance issues (de Andrade and Hunter, 2016), in transgenic plants there is a continuous supply of dsRNA, which increases the selection pressure and favors resistance development in the population. The development of RNAi resistance may be related to a reduction in cellular uptake (Khajuria et al., 2018), mutations in mRNA, production of RNAi suppressors (Zheng et al., 2005), upregulation of the target gene or downregulation of the silencing machinery genes (Garbutt and Reynolds, 2012), increased nuclease activity (dsRNases) (Spit et al., 2017), or even behavioral changes. However, when non-transformative delivery techniques are adopted, insects and pathogens have limited exposure to the dsRNA molecules due to the transient feature of such molecules, preventing the development of resistance in the target organisms.

Non-transformative delivery methods can be developed for use on several crops, targeting pests in different regions. Although GM event approval is more complicated, RNA-based non-transformative products will also undergo regulation procedures, although they will probably be less complicated and time-consuming than for GM plants. Also, an important aspect related to the legislation of non-transformative products is that RNA-based biopesticides will probably need to be approved in only the producing country, unlike GM plants, which needs approval in both import and export countries.

## Successful Non-Transformative Delivery Cases

Based on the advances made in the last decades regarding the use of RNAi in crop protection, it is believed that this technology will soon reach growers as dsRNA/siRNA-based products (Cagliari et al., 2018; Mat Jalaluddin et al., 2018). The application of RNAs targeting essential insect or fungi genes can significantly impair growth, increase mortality rate, and, in some cases, suppress insecticide/fungicide resistance (Pridgeon et al., 2008; Killiny et al., 2014). Although RNAi is not currently functional in every delivery method and every insect life stage or target gene (San Miguel and Scott, 2016), this technology has great potential, especially for insects and diseases with high insecticide- and fungicide-resistance problems.

On the development of non-transformative delivery technologies, in 2011 the Monsanto company published the patent WO 2011/112570 in which the company uses sprayable polynucleotide molecules to regulate gene expression in plants (Sammons et al., 2011). According to the patent, dsRNAs, siRNAs, and even single-stranded DNA oligonucleotides triggered efficient local and systemic silencing of *N. benthamiana* endogenous genes. However, in another experiment, researchers were unsuccessful in inducing gene silencing in plants through siRNA application, including spraying, syringe injection, or siRNAs infiltration, yet they achieved success through high-pressure spraying of siRNAs (Dalakouras et al., 2016).

The delivery system varies according to the target organism and crop (**Table 1**). The selection of the delivery strategies (*i.e.*, foliar sprays, irrigation, trunk injection, and baits among others) is the first step to achieve good control results, determining the success of the technology usage. The correct choice of delivery system will expedite the entire process and save years of development and commercialization (de Andrade and Hunter, 2016). Hence, the main non-transformative delivery methods and their applications in insect and disease management, shown in **Table 3**, will be discussed further in the following sections.

**Table 3:** Non-transformative delivery strategies for insects, pathogens, and virus management.

Target pest	Crop	Delivery strategy	Target gene		Molecule	Size	Molecule concentration	Results	Reference	
	Insects									
*Plutella xylostella*	Kale	Foliar spray	AChE2		siRNA	18–27 bp	200 µg/ml	Approximately 60% mortality.	(Gong et al., 2013)	
*Leptinotarsa decemlineata*	Potato	Foliar spray	Actin		dsRNA	50 – 297 bp	5 μg leaf ^−1^	Significant mortality in dsRNA length-depend pattern.	(San Miguel and Scott, 2016)	
*Diaprepes abbreviates*	Citrus	Foliar spray	Not informed		dsRNA	Not informed	Not informed	Control started 4-5 days after dsRNA application.	(de Andrade and Hunter, 2016)	
*Diaphorina citri*;	Citrus approximately 2.5 m tall and Grapevines	Trunk injection; root drench	Arginine kinase		dsRNA	Not informed	2 g in 15 liters of water	Insects successfully uptake dsRNA from the treated plants; dsRNA was detected in plants for at least 57 days.	(Hunter et al., 2012)	
*Bactericera cockerelli*;										
*Homalodisca vitripennis*										
*Nilaparvata lugens*	Rice	Roots soaking	Ces		dsRNA	Not informed	1 mL (1.0 mg mL^−1^ of water)	Gene knocked down; nymph mortality.	(Li et al., 2015)	
			CYP18A1							
*Ostrinia furnacalis*	Maize	Irrigation	KTI		dsRNA		10 mL (0.5 mg mL^−1^ water)	Gene knocked down; larval mortality.		
*Myzus persicae*	Tomato	Foliar application	ZYMV HC-Pro		dsRNA	588 bp	10.5 µg dsRNA in 10 µL water	Insect successfully uptake dsRNA; the dsRNA was processed into siRNA by the insect RNAi machinery.	(Gogoi et al., 2017)	
*Tetranychus urticae*										
*Trialeurodes vaporariorum*								Low dsRNA uptake; No siRNA in insects.		
*Halyomorpha halys*	Green beans	Soaking	JHAMT		dsRNA	200-500 bp	300 µl (0.017 μg μL^-1^ of water)	Significant reduction in gene expression.	(Ghosh et al., 2017)	
			Vg				300 µl (0.067 μg μL^-1^ of water)			
*Planococcus citri*	Tobacco	VIGS using recombinant TMV	Actin		siRNA	Not informed	–	Crawlers feed on recombinant TMV-infected plants showed lower fecundity and pronounced death.	(Khan et al., 2013)	
			CHS1							
			V-ATPase							
*Bactericera cockerelli*	Tomato	VIGS using recombinant TMV	Actin		siRNA	21 nt	–	Gene knocked down in insects feed on these plants; Insects fed on infected tomatillo plants showed a decreased progeny production.	(Wuriyanghan and Falk, 2013)	
	Tomatillo									
	Tobacco									
*Diaphorina citri*	Citrus	VIGS using recombinant CTV	Awd		siRNA	20-22 nt	–	Adults showed malformed-wing phenotype and increased mortality.	(Hajeri et al., 2014)	
*Phenacoccus solenopsis*	Tobacco	VIGS using recombinant PVX	Bur		siRNA	–	–	Insects fed on treated plants showed physical deformities or died.	(Khan et al., 2018)	
			V-ATPase							
*Drosophila melanogaster*	–	VIGS using recombinant FHV; microinjection	RPS13		siRNA	–	–	Significantly higher mortality in insects.	(Taning et al., 2018)	
			Vha26							
			Alpha COP							
*Helicoverpa armigera*	–	dsRNA expressed in bacteria, using recombinant *E. coli* strain HT115; artificial diet coated with engineered bacteria	AK		dsRNA	379-426 bp	30 µL (10^9^ cells)	Knocked down the target gene caused drastic reductions in body weight, body length, and pupation rate, resulting in high mortality.	(Ai et al., 2018)	
*Spodoptera exigua*	Chinese cabbage	dsRNA expressed in bacteria, using recombinant *E. coli* strain HT115	INT		dsRNA	410 bp	10^7^ cells per larva	Significant reduction of the SeINT expression resulting in insect mortality; Pretreatment with an ultra-sonication increased the insecticidal activity of the recombinant bacteria, and treated larvae became s susceptible to Cry toxin.	(Kim et al., 2015)	
	–	dsRNA expressed in bacteria, using recombinant *E. coli* strain HT115; artificial diet containing engineered bacteria	CHSA		dsRNA	635 bp	High dose (250X), medium dose (50X), and low dose (10X) based on the dilution factors.	Significant reduction in survival rates. Levels of target gene expression, tissue structure, and survival rates were dose-dependent.	(Tian et al., 2009)	
*Lymantria dispar*	–	dsRNA expressed in bacteria, using recombinant *E. coli* strain HT115; diet with engineered bacteria	Locus 365		dsRNA	–	300 μl of bacteria culture	Target-gene knocked down, reduction in body mass and egg masses.	(Ghosh and Gundersen-Rindal, 2017)	
			Locus 28365							
*Mythimna separata*	–	dsRNA expressed in bacteria, using recombinant *E. coli* strain HT115; artificial diet containing engineered bacteria	Chi		dsRNA	700 bp	–	Target gene knocked down after oral delivery of engineered bacteria, resulting in resulted in increased mortality and reduction in body weight of the feeding larvae.w	(Ganbaatar et al., 2017)	
*Bactrocera dorsalis*	–	dsRNA expressed in bacteria, using recombinant *E. coli* strain HT115; artificial diet containing engineered bacteria	Rpl19		dsRNA	–	200 ml 250X of bacteria culture expressing dsRNA.	Successful gene silencing of the target genes after insects were fed on a diet containing engineered bacteria. An over-expression of the target genes after continuously supply of engineered bacteria was also observed.	(Li et al., 2011)	
			V-ATPase							
			Rab11							
			Noa							
*Bemisia tabaci*	Hibiscus	dsRNA expressed in fungus, using engineered *Isaria fumosorosea*	TLR7		dsRNA	548 bp	2x10^7^,1x10^7^,5×10^6^, 2.5x10^6^ spores mL^-1^	The engineered IfB01-TRL7 strain increased the mortality of whitefly nymphs compared to the IfB01 strain. The IfB01-TRL7 strain also show higher virulence, with decreased and shortened values of LC50 and LT50.	(Chen et al., 2015)	
*Manduca sexta*	Tobacco	VIGS using recombinant TRV	DCL1	In tobacco plants	dsRNA	≥ 300 bp	–	Knocked down of the DCL target genes in engineered tobacco plants to express a 312 bp fragment of *Ms*CYP6B46 gene increased the gene silencing results.	(Kumar et al., 2012)	
			DCL2							
			DCL3							
			DCL4							
			CYP6	In tobacco hornworm						
**Diseases**										
*Fusarium graminearum*	Barley	Foliar spray	CYP3		dsRNA	791 bp	500 μL (20 ng μL^-1^ of water)	Inhibition of fungal growth.	(Koch et al., 2016)	
*SCMV*	Maize	Bacterial crude extract foliar spraying (*E. coli* strain HT115)	CP		dsRNA	147-247 bp	One-half diluted extraction crude	Inhibition of SCMV infection.	(Gan et al., 2010)	
*Botrytis cinerea*	Tomato, Strawberry, Grape, Lettuce, Onion, Rose	Foliar application	DCL1		sRNA	21-24 nt	400 µl (20 ng µL^–1^)	Both sRNA and dsRNA were uptake by the fungus resulting in fungal growth inhibition.	(Wang et al., 2016b)	
					dsRNA	252 bp				
			DCL2		sRNA	21-14 nt				
					dsRNA	238 bp				
*Sclerotinia sclerotiorum*	Canola	Foliar spray	59 target genes		dsRNA	200-450 bp	10–25 µL of 200–500 ng dsRNA plus 0.02–0.03% Silwet L-77.	From the 59 dsRNAs tested, 20 showed antifungal activity with a reduction in lesion size ranging from 26–85%.	(McLoughlin et al., 2018)	
*Botrytis cinerea*										
BCMV	Tobacco; cowpea	Foliar spray	Nib		dsRNA naked or loaded onto LDH	480 bp	100 μg of in a 1 mL or 250 ng of dsRNA.	Plants were protected from aphid-mediated virus transmission.	(Worrall et al., 2019)	
			CP			461 bp				
Fusarium asiaticum	Wheat	Foliar spray	Myosin 5		dsRNA	496 bp	0.1 pM	Reduced pathogen sensitivity to phenamacril with a reduction in infection.	(Song et al., 2018)	
PPV	Tobacco	Bacterial crude extract foliar spraying (*E. coli* strain HT115)	IR 54		hpRNA	977 bp	Dilution series (1/2 to 1/20) using 3 µg of total nucleic acid/µl.	Dilutions of 1/10 or less did not display disease symptoms upon completion of their life cycles	(Tenllado et al., 2003)	
PMMoV			HC; CP		dsRNA	1492 bp; 1081 bp	One-half diluted French Press preparations derived from engineered bacteria.	Plants treated with dsRNA-expressing preparations showed no virus symptoms (HC: 82% or CP: 73%).		
TMV	Tobacco	Bacterial crude extract foliar spraying (Different *E. coli* strain tested)	CP		dsRNA	480 bp	One-half diluted French Press preparations derived from engineered bacteria.	M-JM109 or M-JM109lacY strains and the pGEM-CP480 vector exhibited the best results producing great quantities of dsRNA. Tobacco plants sprayed with dsRNA crude bacterial extract showed inhibition in TMV infection.	(Yin et al., 2009)	
PMMoV	Tobacco	Foliar spray	RP		dsRNA naked or loaded onto LDH	977 bp	125 µL per cm^2^ (1.25 µg of dsRNA and/or 3.75 µg of LDH).	Virus protection for at least 20 days.	(Mitter et al., 2017a)	
CMV	Cowpea		2b supressor			330 bp				
*Fusarium asiaticum.*	Wheat	Foliar spray after leaves were wounded using quartz sand	β2-tubulinX		dsRNA	480 bp	40 ng μL^−1^ of water	Antifungal activity against these fungi with a reduction in the dosage of carbendazim fungicides necessary to control the pathogens.	(Gu et al., 2019)	
*Botrytis cinerea*	Cucumber									
*Magnaporthe oryzae*	Barley									
*Colletotrichum truncatum*	Soybean									

AChE2, acetylcholine esterase; CP, Coat Protein; Ces, carboxylesterase; ZYMV, Zucchini yellow mosaic virus; JHAMT, Juvenile hormone acid O-methyltransferase; Vg, Vitellogenin; CYP: cytochrome P450; KT, Kunitz-type trypsin inhibitor; DCL, Dicer-like; BCMV: Bean common mosaic virus; PMMoV, Pepper mild mottle virus; CMV, Cucumber mosaic virus; LDH, double-layered hydroxide; RP, Replicase; CTV, Citrus tristeza virus; Awd, abnormal wing disc; BUR, Bursicon; FHV, Flock house virus; RPS13, Ribosomal protein S13; Vha26, Vacuolar H[+]-ATPase 26kD E subunit; Alpha COP, Alpha-coatomer protein; AK, Arginine kinase; INT, *β*1 integrin gene; CHSA, Chitin synthase gene A; Chi, chitinase; Rpl19, ribosomal protein Rpl19; Sec23, Protein transport protein sec23; vATPaseE, Vacuolar ATP synthase subunit E; vATPaseB, Vacuolar ATP synthase subunit B; COP*β*, Coatomer subunit beta; SCMV, Sugarcane Mosaic Virus; HC, Helper component; IR, replicase; TLR7, Toll-like receptor 7; LC50, Lethal Concentration 50; LT50, Lethal Time 50; VIGS, Virus-induced gene silencing.

### Foliar Application

For pests feeding/growing on stems, foliage, or fruit/seeds, foliar spraying may be an alternative for the delivery of RNA molecules. Thus, the RNA-based formulations are evaluated similarly to topical insecticides where the RNA solution is sprayed on leaves, fed to the target insects, and the effects are observed (de Andrade and Hunter, 2016). Due to the chemical properties of RNAs, a short half-life is expected compared to chemical pesticides. Sprayable RNAs would therefore be an environmentally friendly alternative to synthetic pesticides (Fire and Won, 2013; Wang and Jin, 2017).

One of the first studies exploring the applications of sprayable RNA molecules to control insect pests was conducted using siRNA molecules against the diamondback moth, *Plutella xylostella*. Mortality rates of ∼60% were observed when larvae were fed with *Brassica* spp. leaves sprayed with chemically synthesized siRNAs targeting the *acetylcholine esterase* genes *AchE2* (Gong et al., 2013). In an attempt to control the Colorado potato beetle, *Leptinotarsa decemlineata*, foliar application of naked dsRNA targeting the *actin* gene was sufficiently stable for at least 28 days under greenhouse conditions, resulting in significant insect control (San Miguel and Scott, 2016). The same strategy was tested with the aim to control the xylem-feeding leafhopper (*Homalodisca vitripennis*), the phloem-feeding Asian citrus psyllid (*Diaphorina citri*) (Hunter et al., 2012), and the Diaprepes root weevil (*Diaprepes abbreviates*) on citrus leaves, showing a promising alternative to control these insects (de Andrade and Hunter, 2016). In tomato leaves gently rubbed with dsRNA solution, the molecules were rapidly absorbed by tomato plants and were taken up by aphids (*Myzus persicae*), mites (*Tetranychus urticae*), and in fewer numbers, whiteflies (*Trialeurodes vaporariorum*) (Gogoi et al., 2017). Hence, siRNA molecules were only detected in tomato plants, aphids and mites, and they were absent in the whiteflies, in which the dsRNA amounts did not reach the threshold necessary to induce RNAi machinery.

The use of RNAs in foliar application to manage pathogen infection and resistance in crops was also explored. In 2013, scientist discovered that Dicer-like protein 1 and 2 from *Botrytis* (Bc-DCL1; Bc-DCL2) fungus produces small RNAs (Bc-sRNAs), which are delivered into plant cells, silencing host immunity genes (Weiberg et al., 2013). Years later, researches applied siRNAs and dsRNAs targeting *Botrytis cinerea* DCL1 and DCL 2 (Bc-DCL1/2) onto the surface of fruits (tomato, strawberry, and grape), vegetables (lettuce and onion), and flowers (roses), which resulted in the significant inhibition of grey mold disease development (Wang et al., 2016b). In both cases, naked dsRNA/siRNA treatment was able to protect plants from the microbial pathogen for up to ten days after spraying. Moreover, these researchers showed that plants infected with another pathogen, *Verticillium dahlia*, displayed severe wilt disease symptoms, indicating that Bc-DCL1/2 RNAs were specific to *B. cinerea* DCL genes and did not cause non-target effects (Wang et al., 2016b). In the same year, a breakthrough work showed the foliar application of dsRNA targeting the *cytochrome P450* (*CYP3*) gene in *F. graminearum*, resulting in the successful inhibition of fungal growth in local directly sprayed leaves as well as the distal non-sprayed leaves in barley plants (Koch et al., 2016). DsRNA foliar applications also conferred protection against *Sclerotinia sclerotiorum* and *B. cinerea* in *Brassica napus* (McLoughlin et al., 2018). Due to the relative ease of design and the high specificity and applicability to a wide range of pathogens, the use of “RNA fungicides” as anti-fungal agents offers unprecedented potential as a new plant protection strategy that is also less harmful to the environment.

Furthermore, the use of RNA to target pathogen resistance to regular fungicides is also under development. Spraying wheat plants with dsRNA targeting the *Fusarium asiaticum* *myosin 5* gene resulted in increased pathogen sensitivity to phenamacril with a reduction in infection (Song et al., 2018). Although dsRNA has a high specificity, it is also possible for dsRNA molecules to target a specific group. DsRNA molecules of a *β2-tubulin* gene derived from *F. asiaticum* suppressed the fungal activity of *F. asiaticum*, *B. cinerea*, *Magnaporthe oryzae*, and *Colletotrichum truncatum* in wheat, cucumber, barley, and soybean, respectively (Gu et al., 2019). Alongside this, the dsRNA molecule also functioned to reduce the dosage of carbendazim (MBC) fungicide to control the pathogens. Thus, the combination of dsRNA and site-specific fungicide can be a control strategy against resistant pathogen infection in the field, rather than the individual use of dsRNA or fungicides.

Co-inoculation of synthesized dsRNA to protect plants against a virus/viroid is effective at preventing virus infection in a range of plants through mechanical inoculation, thereby increasing the prospect for foliar dsRNA application in virus management in plants (Tenllado and Díaz-Ruíz, 2001; Carbonell et al., 2008; Šafářová et al., 2014; Konakalla et al., 2016). Recently, Niehl et al. (2018) suggested the term “plants vaccines,” citing the use of sprayable dsRNA to control the *Tobacco mosaic virus* (TMV) in tobacco, similarly to vaccines for animals that use dead or living (but weakened) microorganisms. These researchers used fragments of the virus’ genetic material to produce the “vaccines” (dsRNA) together with the plant’s immune system as a defense mechanism. This system opens a range of opportunities for the use of RNAi in a non-transformative approach in the control of viruses in crops.

The potential applications of SIGS for plant protection have had significant improvement due to the recent advances in nanoparticle technology. To overcome problems related to dsRNA stability, a double-layered hydroxide (LDH) nanoparticle was developed and combined with dsRNA molecules to yield “BioClay” (Mitter et al., 2017b). The clay nanoparticles are positively charged and thus bind and protect the negatively charged dsRNAs; delivery occurs when atmospheric carbon dioxide and moisture reacts with the clay nanoparticles, breaking the LDH and gradually releasing the dsRNAs. Using the dsRNA-LDH complex, researchers were able to achieve long-term gene silencing results by protecting tobacco plants from a virus for up to 20 days with a single spray, extending the period from five to seven days using naked dsRNA (Mitter et al., 2017a; Mitter et al., 2017b). In another experiment, researchers sprayed tobacco and cowpea plants with BioClay nanosheets of dsRNA from the coat protein from the *Bean common mosaic virus* (BCMV) five days before exposure to viruliferous aphids (Worrall et al., 2019). The researchers found that BioClay molecules protected plants from BCMV infection due to aphid-mediated virus transmission and considered this an important step toward the development of a practical application of dsRNA in crop protection. These results using sprayable dsRNA are encouraging, and although more progress is needed on several fronts, RNA-based biopesticides are expected to reach the market soon. Monsanto is developing the use of RNAi through a technology called “BioDirect,” in which dsRNA formulation is applied exogenously to protected plants against insect and pathogen attack (https://monsanto.com/innovations/agricultural-biologicals/). Syngenta scientists are also developing lines of biocontrol products based on RNAi to protect potato plants from the attack of the Colorado potato beetle (https://www.youtube.com/embed/BiVZbAy4NHw?ecver=1). These technologies will help growers to improve pest control in crops, resulting in increased yields and improved quality.

### Trunk-Injection

The use of trunk injection to deliver dsRNA to control insects has been tested and showed great progress, especially in perennial plants such as citrus. Developed citrus plants (2.5 meters tall) and grapevines were treated with 2 g of dsRNA in 15 L of water solution applied by root drench and injection into the trunk, and dsRNA was taken up into whole plant systems over three months (Hunter et al., 2012). In citrus plants, the dsRNA was detected in the psyllid and the spittlebug from five to eight days after entering the plants, allowing the development of pest suppression.

Recently, researchers showed that hairpin RNAs (hpRNAs) and siRNAs delivered through petiole absorption or trunk injection to *M. domestica* and *V. vinifera* plants were restricted to the xylem vessels and apoplast, being efficiently translocated (Dalakouras et al., 2018). Due to this characteristic, the plant Dicer-like (DCL) endonucleases were unable to process the hpRNA. Injected RNA molecules were thus detected in plants for at least ten days post-application. However, when siRNA was delivered to *N. benthamiana* through petiole absorption, the molecules were not efficiently translocated. These innovative methods may have a significant impact on pest management against chewing or xylem sap-feeding insects and eukaryotic pathogens that reside in the xylem, allowing an essay reposition of the RNA-based solution and efficient plant protection for a longer period.

### Irrigation

Hunter and collaborators showed that the dsRNA applied through a root drench in adult citrus plants (2.5 m tall) could effectively control psyllids and leafhoppers for up to 57 days (Hunter et al., 2012). They were able to detect the RNA molecules in the citrus plants for over three months. Rice plant roots soaked in a solution containing dsRNA targeting *carboxylesterase* (*Ces*) and *CYP18A1* genes from the brown planthopper (BPH), *Nilaparvata lugens*, significantly knocked down these genes, resulting in high mortality when BPH nymphs were fed on treated plants (Li et al., 2015). This study also showed maize seedlings irrigated with dsRNA of the Kunitz-type trypsin inhibitors (dsKTI) from the Asian corn borer (ACB), *Ostrinia furnacalis*, and this resulted in high larval mortality rates. Recently, Ghosh and collaborators showed that *Halyomorpha hayls* nymphs fed on green beans soaked in dsRNA solution targeting *JHAMT* (*Juvenile hormone acid O-methyltransferas*) and *Vg* (*Vitellogenin*) genes resulted in a significant reduction in gene expression, indicating that RNAi can be efficiently employed through vegetable delivery in plant-sap-feeding insects (Ghosh et al., 2017). The delivery of gene silencing molecules through irrigation can be an alternative for crops that use irrigation in the normal growing system, allowing for the continuous supply of RNA molecules. However, Dubelman et al. (2014) reported short persistence of dsRNA molecules in the soil, with a rapid breakdown within 2–3 days. Therefore, the dsRNA stability in the soil is still an issue affecting RNAi efficiency (Joga et al., 2016), and the feasibility of this delivery strategy relies on the advances of formulations to protect RNA molecules from degradation.

### Microbe-Induced Gene Silencing

Many microbes such as viruses, bacteria, yeasts, and fungi can be engineered to generate a vector for RNAi induction through the continuous production of dsRNA into the host, and this is being considered as a promising dsRNA delivery method for insect and disease management (Fjose et al., 2001; Whitten et al., 2016; Cagliari et al., 2018; Dubrovina and Kiselev, 2019; Goulin et al., 2019).

Virus-induced gene silencing (VIGS) is a naturally occurring and very effective defense system that is consistent with the normal dynamics of host–pathogen interactions and which is widely harnessed as a powerful tool for the study of gene function in plants (Ratcliff et al., 1997; Waterhouse et al., 2001; Lu et al., 2003; Robertson, 2004; Baulcombe, 2015). VIGS is transiently transformative and does not cause alterations in the plant’s genetic composition, unlike stable RNAi and mutant plants. Furthermore, VIGS can be transmitted to plant progeny and actively co-opts the plant for expression of dsRNA (Senthil-Kumar and Mysore, 2011). Moreover, VIGS enables high throughput screening of potential targets genes to control insect pest (Gu and Knipple, 2013; Nandety et al., 2015; Kolliopoulou et al., 2017). In Lepidoptera, three midgut-expressed *CYP* genes in *Manduca sexta* were targeted through the engineering of *Tobacco Rattle Virus* (TRV) for dsRNA delivery in *Nicotiana attenuata* (Kumar et al., 2012). Also, plant-virus based dsRNA delivery vectors are promising tools for targeting phloem-feeding insects because almost all plant-infecting viruses infect and move systemically *via* the phloem (Nandety et al., 2015). To demonstrate this, researchers used a recombinant TMV to express RNAi effectors in *N. benthamiana* plants against the citrus mealybug (*Planococcus citri*) and observed lower fecundity and a pronounced death of crawlers after feeding on recombinant TMV-infected plants (Khan et al., 2013). Similarly, infecting tomatillo (*Physalis philadelphica*) plants with recombinant TMV-expressing RNAi effectors also resulted in a decrease in *Bactericera cockerelli* progeny production after feeding (Wuriyanghan and Falk, 2013). In another study, researchers engineered *Citrus tristeza virus* (CTV), a common virus of citrus, with *D. citri* truncated *abnormal wing disc* (*awd*) RNA sequence triggering *awd* gene silencing after *D. citri* nymphs fed on infected plants, causing wing malformation and mortality in adult insects (Hajeri et al., 2014). The *Potato virus X* (PVX) engineered with *Bursicon* and *V-ATPase* gene sequences significantly reduced the population of the cotton mealybug (*Phenacoccus solenopsis*) after insects fed on *Nicotiana tabacum* plants inoculated with the recombinant PVX (Khan et al., 2018). Furthermore, insect-specific viruses can be exploited as VIGS vectors to control insect pests (Kolliopoulou et al., 2017; Nouri et al., 2018). For instance, researchers investigated the ability of engineered *Flock house virus* (FHV) to induce gene suppression through RNAi in S2 cells derived from *D. melanogaster* embryos and insects at the adult stage. The recombinant FHV carrying the target gene sequences caused significantly higher mortality (60–73% and 100%) than the wild type virus (24 and 71%) in both S2 cells and adult insects, respectively (Taning et al., 2018).

To date, the sources of RNA-based molecules (dsRNA or siRNA) commonly utilized in insect and disease management studies are costly synthetic molecules or are produced through time-consuming, laborious procedures. To overcome the shortages of these methods, the potential of delivering dsRNA expressed in bacteria has been investigated, providing an alternative method for large-scale target gene screening (de Andrade and Hunter, 2016; Zotti et al., 2017). In Lepidoptera, the cotton bollworm (*Helicoverpa armigera*) larvae exposed to an artificial diet coated with engineered bacteria for five days showed high mortality and inhibition in the expression levels of target genes, causing drastic reductions in body weight, body length, and pupation rate (Ai et al., 2018). Oral toxicity of *Escherichia coli* expressing dsRNA targeting the *integrin β1 subunit* was observed in *Spodoptera exigua* larvae; this resulted in insect mortality, damage to the midgut epithelium tissue, exhibition of a marked loss of cell-cell contact, and remarkable cell death, which further resulted in increased susceptibility to a Cry insecticidal protein from *B. thuringiensis* (Kim et al., 2015). Also, the growth and development of *S. exigua* larvae fed with *E. coli* expressing dsRNA targeting *chitin synthase A* was disturbed, resulting in mortality (Tian et al., 2009). Moreover, in the gypsy moth (*Lymantria dispar*), a serious insect pest of the North American forests, bacterial expression of dsRNA resulted in target-gene knockdown and a subsequent reduction in body mass and egg masses (Ghosh and Gundersen-Rindal, 2017). In the oriental armyworm (*Mythimna separate*), a study showed that oral delivery of bacterially expressed dsRNA led to RNAi effects, with knockdown of target genes, reduction of body weight, and increased mortality (Ganbaatar et al., 2017). In Diptera, *Bactrocera dorsalis* adults fed on an artificial diet coated with *E. coli* expressing dsRNA exhibited a reduction in target gene mRNA levels and a reduction in egg-laying (Li et al., 2011). In Coleoptera, the potential of feeding dsRNA expressed in bacteria to manage populations of Colorado potato beetle (*L. decemlineata*) was observed due to the resulting knockdown of five target genes tested, which caused significant mortality and reduced body weight gain in treated beetles (Zhu et al., 2011).

Besides the use of bacteria as a dsRNA delivery method to pests, these microorganisms have been used to produce large amounts of dsRNAs, which can be sprayed on crops at any time with lower costs (Joga et al., 2016). For example, the *E. coli* HT115 (DE3) strain has been used to produce large amounts of dsRNA since it lacks the enzyme that degrades dsRNAs (Papic et al., 2018; Ahn et al., 2019). Also, studies have shown the efficiency of dsRNA produced in bacteria to control plant viruses (Robinson et al., 2014; Mitter et al., 2017b). Crude extracts of *E. coli* HT115 containing dsRNA targeting the *Sugarcane mosaic virus* (SCMV) *coat protein* gene were used in maize plants as a preventive spray and they inhibited the SCMV infection (Gan et al., 2010). Other works reported the use of bacteria to produce dsRNAs from *Pepper mild mottle virus* (PMMoV), PPV, and TMV to protect plants against these pathogens. The application of crude bacterial preparation via spray onto tobacco plant surfaces provided protection against infection from these viruses (Tenllado et al., 2003; Yin et al., 2009). Moreover, this system of dsRNA production in bacteria can deliver multiple virus dsRNAs to disrupt several virus species at once and may achieve multiple virus resistances at one time (Tenllado and Díaz-Ruíz, 2001; Yin et al., 2009).

Recently, advances in sequencing technology and the characterization of insect gut microbiota are leading to the identification of novel symbiotic microorganisms suitable to be genetically modified and used as dsRNA delivery vectors to control insects (Krishnan et al., 2014). Using symbiont-mediated RNAi is an intriguing strategy in which the relationship between culturable symbiotic gut bacteria, or yeast, and the host can be exploited in order to constitutively produce dsRNA to induce RNAi in the host, and the use of symbiotic bacteria has been shown to be a promising delivery strategy to control insects (Abrieux and Chiu, 2016; Joga et al., 2016; Whitten and Dyson, 2017). Also, dsRNA can be delivered into target pests through the infection of entomopathogenic fungus and may result in the development of a new RNAi methodology for pest control. For instance, the application of *Isaria fumosorosea*, a common fungal pathogen of the B-biotype *Bemisia tabaci*, expressing dsRNA of whitefly immunity-related genes, resulted in knockdown of the target gene and increased whitefly mortality (Chen et al., 2015).

Although viruses and bacteria, following genetic modification to express dsRNA and induce gene silencing, are promising strategies to deliver dsRNA in the field, they will be considered as GM products and will suffer the same regulatory and public acceptance obstacles as GM crops.

### Other Applications

In relation to the natural role of RNAi to protect cells from virus infections, this technology could be used to protect beneficial insects, such as bees, from viral diseases. In 2010, large-scale field trials tested the efficiency of Rembee™ (Beeologics, LLC, Miami, FL, USA), a dsRNA product designed to protect honeybees (*Apis mellifera*) from *Israeli acute paralysis virus* (IAPV) infection (Hunter et al., 2010). The product successfully protected the hives from the virus infection, resulting in several bees that were twice as large in the dsRNA-treated hives compared to untreated. As a result, dsRNA-treated hives produced three times as much honey compared to untreated ones. In another study, a similar result was observed in bumblebees (*Bombus terrestris*), which upon being fed on IAPV virus-specific dsRNAs, showed decreased mortality (Piot et al., 2015). In other studies carried out on *A. mellifera*, RNAi was also efficient against the internal microsporidian parasite *Nosema* (Paldi et al., 2010; Rodríguez-García et al., 2018) and the obligatory ectoparasite *Varroa destructor* (Garbian et al., 2012). The control of these organisms, which are associated with colony decline, improved the health of hives and shines a light on the development of effective treatment alternatives for diseases in bees and other beneficial insects in the future.

## Issues Involving Non-Transformative Delivery Approaches

In the near future, the exogenous application of RNA molecules to induce RNAi-mediated gene silencing will influence the traditional way we protect crops from insects and pathogens. Due to uptake restrictions, it is believed that the development of RNA-based products will focus on the use of dsRNA as the molecule to induce gene silencing (Sammons et al., 2011). The minimum required length of a dsRNA to achieve an RNAi effect will vary depending on target genes and species (Bolognesi et al., 2012). Consequently, the formulations can contain only one dsRNA molecule, be a combination of short and long dsRNAs targeting one or more genes, or otherwise be a combination of dsRNA and insecticide or fungicide, managing a resistant population and reaching better results.

Under field conditions, RNA-based biopesticides would need periodical applications following plant growth to ensure plant protection. Also, while the RNA-based products are a new and highly specific mode of action, the timing issues of “when should I spray?”, a dilemma that growers already have with current chemical control approaches, is also something that needs to be studied and understood. Although the vascular system of plants translocate RNAs (Melnyk et al., 2011), allowing RNA molecules to travel across long distances inside the plant and protecting untreated areas, the necessity of reapplication implies an increase in cost. Thus, it is expected that, with the use of non-transformative strategies to control insects and pathogens, the dsRNA molecule will remain active long enough to effectively control the target pest. Moreover, although selection of the most effective target gene is desirable, even partial suppression can cause severe damage and irreversible lethal effects (Huvenne and Smagghe, 2010). Transient effects of this technique should not be an overwhelming drawback to the use of non-transformative approaches. In addition to this, the development of more efficient dsRNA mass production systems will reduce costs and, together with the release of new formulation strategies, will allow foliar spray, trunk injection, and irrigation, among other approaches, to be exploited as potential control strategies (Hunter et al., 2012; de Andrade and Hunter, 2016).

DsRNA production costs have been dropping significantly over the last years, from ∼ $12,500 USD per gram in 2008 to less than $60 USD per gram in 2018 (Cagliari et al., 2018), with an expectation of further significant reduction in prices in the next years. Mass dsRNA production systems, such as *in vitro* or *in vivo* production systems, allow high dsRNA production with the reduction in costs. These are strategies based on the hybridization of two single-stranded RNAs (ssRNAs), enzymatically synthesized, which can be performed *in vitro* (Tenllado and Díaz-Ruíz, 2001; Koch et al., 2016; Konakalla et al., 2016; Wang et al., 2016b) or *in vivo* (using bacterial cells deficient of enzyme RNase III that degrades dsRNAs) (Tenllado et al., 2003; Gan et al., 2010). Although an *in vivo* system allows for the production of bulk amounts of dsRNA compared to *in vitro* synthesis, it still results in high cost, hard purification, and high labor demand (AgroRNA, http://www.agrorna.com/sub_02.html), and, after all, is still naked dsRNA that under field conditions presents a shorter half-life. Thus, dsRNA formulation is a promising alternative to increase stability and boost the efficiency of gene silencing in recalcitrant species in Lepidoptera and Hemiptera, allowing plants to be protected for longer.

The technology “BioClay,” a layered double hydroxide (LDH) clay nanosheet, provided high dsRNA stability under field conditions, increasing the residual period of dsRNA on plants and protecting them from virus infection for up to 30 days compared to naked dsRNA (Mitter et al., 2017a). Guanylate Polymers increased RNAi efficiency in *S. exigua* (Christiaens et al., 2018b) and *Spodoptera frugiperda* (Parsons et al., 2018), and they pave the way for future applications of RNA-based pest control strategies in lepidopteran insects. This technology is based on the use of formulations to enhance stability of dsRNA in insects. Encapsulation of dsRNA molecules in liposome complexes also increased dsRNA stability and enhanced cellular uptake in Dipteran insects (Whyard et al., 2009; Taning et al., 2016) and Blattodea (Lin et al., 2017). In *Euchistus heros*, liposome complexes increased nymph mortality compared to naked dsRNA (Castellanos et al., 2018). However, in some cases, even with the use of formulation the dsRNA molecules were unable to initiate the RNAi process. This was the case in the migratory locust (*Locusta migratoria*), where liposome encapsulation was not efficient to protect the dsRNA, leading to inefficient RNAi in this species (Luo et al., 2013).

Considering the hostile environmental conditions to which dsRNA molecules are exposed in the field, a biotechnology company called RNAagri (former APSE) developed a system where APSE RNA Containers (ARCs) are produced by *E. coli* bacteria, allowing for the mass production of encapsulated ready-to-spray dsRNA (APSE technology; www.apsellc.com). This technology is based on bacteria engineered with a plasmid to produce naturally occurring proteins such as capsids, which are then co-transformed with another plasmid coding for the target dsRNA or siRNA together with a sequence called the “packing site”. The double-transformed *E. coli* are then purified, resulting in self-assemble particles that have encapsulated the desired RNAs. These particles protect the RNAs and enhance resistance to adverse environmental conditions, and, once sprayed, they are expected to be taken up by the insect rapidly (Kolliopoulou et al., 2017). The development of formulations to carry dsRNA efficiently up to the target organism is of paramount importance to the success of developing non-transformative strategies for pest control, and advances in this area in the future will boost the use of these strategies.

Successful cases using foliar spray, irrigation, and trunk injection have already been reported (**Table 3**), but the application range may be much broader. The choice of the dsRNA delivery strategy is of great importance in the development of non-transformative delivery methods, and it will vary according to the target pest and crop. RNAi efficiency naturally varies among the target species, life stage, and delivery strategy, and the choice of a correct combination of these factors will save years of research and resources. Regardless of the delivery strategy or target species, for a successful non-transformative RNAi strategy it is also of paramount importance to identify unique regions in essential target genes so that little changes in expression level will provoke severe consequences. For example, foliar application of dsRNA was unable to induce the RNAi machinery in *T. vaporariorum* due to the low dsRNA uptake by the insects (Gogoi et al., 2017). In order to achieve success using RNAi-based gene silencing as a control strategy, low amounts of RNA molecules need to be enough to trigger the machinery and lead to insect or pathogen mortality. In insects, screening for target genes through artificial diet containing dsRNA is an easy procedure to screen large numbers of dsRNA molecules, resemble field conditions (Araujo et al., 2007; Whyard et al., 2009; Aronstein et al., 2011), and address important issues such as better target genes, effective dsRNA, and effective lethal concentration (LC50) (Araujo et al., 2007; Baum et al., 2007; Bachman et al., 2013). However, under field conditions it is difficult to establish the amount of dsRNA taken up by the target pest, which hinder determination of the LC50.

Coleopteran insects are considered very susceptible to RNAi (Baum et al., 2007; Baum and Roberts, 2014), while insects in the order Lepidoptera are considered recalcitrant and high dsRNA concentrations are required to achieve successful gene silencing results (Terenius et al., 2011). Limiting factors, such as dsRNA degradation (Wang et al., 2016a; Guan et al., 2018) and the entrapment of internalized dsRNA in endosomes (Yoon et al., 2017), have recently been associated with unsuccessful RNAi (Niu et al., 2018). In some hemipteran insects, such as *Acyrthosiphon pisum*, the lack of response under dsRNA supply is also associated with high nuclease activity (Christiaens et al., 2014). Thus, we believe significant advances in dsRNA formulation will occur in the next years, and so the development of RNA-based non-transformative products will be focused on non-recalcitrant groups.

Another important point in the use of non-transformative strategies for RNA delivery, mainly *via* foliar application, is that, during the application, not only the target pest will receive the RNA molecules, but also non-target insects. In GM plants, researchers have shown that expressed dsRNA has a high degree of specificity towards control insects (Dillin, 2003; Whyard et al., 2009; Petrick et al., 2013) or pathogens (Koch et al., 2013). However, other studies have shown that siRNAs can knockdown non-target genes (Birmingham et al., 2006). In mammals, studies have shown that even with differences between the nucleotide sequences from siRNA and the target mRNA gene silencing still occurs (Jackson et al., 2003; Schwarz et al., 2006; Huang et al., 2009). However, there is no consensus among scientists on the number of nucleotides from the siRNA that must match the target sequence identically, and more research is needed to determine if the same issues found in mammalian cells apply to other organisms such as insects or pathogens (Christiaens et al., 2018a). Therefore, target regions and dsRNA molecule design is very important. Baum et al. (2007) tested the specificity of dsRNA molecules based on the identity of the nucleotide sequence of the *V-ATPase* gene *subunits A* and *E* between *D. v. virgifera* and *L. decemlineata*. The target sequences of the *V-ATPase subunit A* shared 83% identity, while the target sequences of the *V-ATPase E* subunit of these insects shared 79% identity. Feeding both *D. v. virgifera* and *L. decemlineata* with the non-specific dsRNAs caused mortality in both species (Baum et al., 2007). However, researchers already expected this response as most of the ∼21 nt siRNAs obtained would have a similarity to both species, causing non-specific silencing. GM tobacco plants expressing a dsRNA targeting the *EcR* gene in *H. armigera* were also effective against another lepidopteran pest, *S. exigua* (Zhu et al., 2012). The target sequence of both species had a high similarity in the nucleotides sequences (89%), and, when both species fed on the GM tobacco plants, this resulted in mortality levels between 40–50%. However, when the necessary care at the time of dsRNA design is taken, it is possible to obtain extremely specific or broad-range molecules. To show the specificity of dsRNA-based gene silencing, the molecules were designed to target the *V-ATPase* gene in four different species, *D. melanogaster* (Diptera), *Tribolium castaneum* (Coleoptera), *A. pisum *(Hemiptera), and *M. sexta* (Lepidoptera), resulting in target gene silencing with no effects over non-target species (Whyard et al., 2009). They also demonstrated the feasibility of designing specific dsRNA molecules even within species of the same genus. Hence, the design of the dsRNA will determine the action spectrum of the molecules; molecules with a larger action spectrum are not necessarily harmful. If carefully designed, broad-spectrum RNA-based molecules can be used to protect plants against diverse insects and pathogens.

## Perspectives in a Global View

During the last decade, significant advances have been made to find better ways to control insects and pathogens in crops, reduce environmental impacts, and improve profits. Scientists have harnessed technologies such as RNAi-based gene silencing to turn off essential genes in target organisms, leading to mortality. Studies using foliar applications, trunk injection, and irrigation have demonstrated the feasibility and efficacy of RNAi-based gene silencing through non-transformative delivery strategies (**Table 3**). Other delivery methods still need to be investigated, such as seed coats or baits. To our knowledge, no studies for the development of RNA-based products as seed coat or powder/granules formulations are available. While the main objective of the seed coat is to protect plants from the attack of insects and pathogens during the initial growth phase, powder/granules formulations could be applied on the soil or substrate surface. Similarly, the use of baits (spray or station) containing RNA is a promising non-transformative delivery strategy that could be developed for pest control, especially in orchards. The bait spray can consist of an attractant mixed with a specific RNA, while bait stations can be containers with sRNA molecules and attractants, which will attract the pest to the bait. These are techniques that can be explored further in the use of RNAi in crop protection.

RNA biopesticides are compounds occurring naturally in the environment and inside organisms and are thus potentially less harmful than synthetic pesticides. These molecules are naturally internalized by eukaryotic organisms, subject to RNAi pathways, and degraded by natural cellular processes. Also, dsRNAs are rapidly degraded when present in water or soil (Dubelman et al., 2014; Albright III et al., 2017; Fischer et al., 2017; Parker et al., 2019), reducing the chances to leave residues in the environment or food products. As with any control method, targeted insects, pathogens, and viruses can develop resistance.

The use of genomic tools will allow the development of technologies such as RNA-based products to increase crop resistance against insects, pathogens, and viruses. Also, the development of RNA formulations will improve RNAi efficiency and field stability. So, these could even replace chemical pesticides in some applications or, when in combination, reduce the use of chemical pesticides at least.

## Author Contributions

DC, ND, GS, and MZ contributed to the conception of the manuscript. DC and ND wrote the first draft. DC, ND, DG, ES, GS, and MZ wrote sections of the manuscript. GS and MZ revised and edited the manuscript. All authors read, contributed critically to the drafts, and approved the final version.

## Funding

DC is a recipient of a scholarship (140733/2017-5) from the National Council for Scientific and Technological Development (CNPq) in Brazil. ES is a recipient of a scholarship from the Coordination for the Improvement of Higher Education Personnel (CAPES) in Brazil. The Foundation Research-Flanders (FWOVlaanderen) in Belgium, the EUCLID project (No. 633999) and the COST (European Cooperation in Science and Technology) under grant agreement No. CA15223 also supported this work.

## Conflict of Interest

The authors declare that the research was conducted in the absence of any commercial or financial relationships that could be construed as a potential conflict of interest.
